# *P. gingivalis* Modulates Keratinocytes through FOXO Transcription Factors

**DOI:** 10.1371/journal.pone.0078541

**Published:** 2013-11-12

**Authors:** Shuai Li, Guangyu Dong, Anastasios Moschidis, Javier Ortiz, Manjunatha R. Benakanakere, Denis F. Kinane, Dana T. Graves

**Affiliations:** 1 Department of Implant Dentistry, Peking University, School and Hospital of Stomatology, Beijing, China; 2 Departments of Pathology and Periodontics, School of Dental Medicine, University of Pennsylvania, Philadelphia, Pennsylvania, United States of America; 3 Department of Periodontology and Oral Biology, Boston University School of Dental Medicine, Boston, Massachusetts, United States of America; University of Southern California, United States of America

## Abstract

*P. gingivalis* is a prominent periodontal pathogen that has potent effects on host cells. In this study we challenged gingival epithelial cells with *P. gingivalis* with the aim of assessing how mRNA levels of key target genes were modulated by *P. gingivalis* via the transcription factors FOXO1 and FOXO3. Primary mono- and multi-layer cultures of gingival epithelial cells were challenged and barrier function was examined by fluorescent dextran and apoptosis was measured by cytoplasmic histone associated DNA. Gene expression levels were measured by real-time PCR with and without FOXO1 and FOXO3 siRNA compared to scrambled siRNA. *P. gingivalis* induced a loss of barrier function and stimulated gingival epithelial cell apoptosis in multilayer cultures that was in part gingipain dependent. *P. gingivalis* stimulated an increase in FOXO1 and FOXO3 mRNA, enhanced mRNA levels of genes associated with differentiated keratinocyte function (keratin-1, -10, -14, and involucrin), increased mRNA levels of apoptotic genes (BID and TRADD), reduced mRNA levels of genes that regulate inflammation (TLR-2 and -4) and reduced those associated with barrier function (integrin beta-1, -3 and -6). The ability of *P. gingivalis* to modulate these genes was predominantly FOXO1 and FOXO3 dependent. The results indicate that *P. gingivalis* has pronounced effects on gingival keratinocytes and modulates mRNA levels of genes that affect host response, differentiation, apoptosis and barrier function. Moreover, this modulation is dependent upon the transcription factors FOXO1 or FOXO3. In addition, a new function for FOXO1 was identified, that of suppressing TLR-2 and TLR-4 and maintaining integrin beta -1, beta -3 and beta -6 basal mRNA levels in keratinocytes.

## Introduction

Human gingival epithelial cells function as an important part of the innate host defense to limit invasion by microorganisms [1]. This is particularly true for the gingiva due to the high bacterial load that is present as a biofilm on adjacent tooth surfaces or that adheres directly to gingival epithelium. Keratinized oral epithelia found on the palate and gingiva express keratin-1 and -10 in the spinous layer, which are markers of well-differentiated cornified epithelial cells [2]. The cornified epithelium contributes to the barrier provided by gingival epithelium [3]. Gingival epithelial cells come into contact with a variety of bacteria. These include well-defined periodontal pathogens such as *P. gingivalis* as well as commensal organisms such as *S. gordonii* [4]. Other bacteria such as *F. nucleatum* are considered to be opportunistic [5] and have been shown to contribute to periodontal bone loss in animal models [6]. Gingival epithelial cells respond to bacteria in a number of different ways and the response in part depends upon the particular bacteria present.

Epithelial cell monolayers have been extensively examined to study the response to bacteria. *P. gingivalis* has been shown to suppress cytokine production and induce apoptosis [7], [8], [9]. In multi-layer cultures it has been shown that *P. gingivalis* invades and penetrates differentiated gingival epithelium [10], [11], [12]. *P. gingivalis* stimulates cell death by apoptosis in monolayer cultures in part by gingipains, but it is controversial whether gingipains represent a predominant mechanism through which cell death is induced [13]. Although there are several reports on the response of primary human gingival epithelial cells to stimulation by oral bacteria, less is known about the response of differentiated multilayer cultures. Three dimensional keratinocyte cultures have stratified cell layers and terminally differentiate when exposed to an air liquid interface as demonstrated by the expression of keratin-1 and -10 [2], [14], [15].

Two members of the forkhead box-O (FOXO) family of transcription factors that have similar binding domains and activity are FOXO1 and FOXO3 [16], [17]. In some instances they have similar cellular activities but in others they do not. These transcription factors have similar DNA-binding domains and mediate expression of key target genes in a number of cell types and participate in various cellular processes ranging from cell cycle arrest to apoptosis [16], [17]. FOXO1 and FOXO3 have been shown to be important in normal and pathologic processes [18], [19]. Despite their importance in endothelial cells and lymphocyte responses [16], [17] relatively little is known about their role in keratinocyte behavior or in the response of these cells to bacteria.

To investigate further the response of oral keratinocytes to *P. gingivalis* we show that *P. gingivalis* disrupts barrier function, induces apoptosis of multilayer gingival epithelial cultures and induces expression of the transcription factors FOXO1 and FOXO3. Moreover, the regulation of several key target genes by *P. gingivalis* is dependent on FOXO1 or FOXO3 including several involved in cell death, barrier function, differentiation and inflammation.

## Methods

### Ethics Statement

Gingival tissue biopsies were obtained with written informed consent from periodontally healthy patients undergoing oral surgical procedure at the University of Pennsylvania's School of Dental Medicine, approved by the University of Pennsylvania Institutional Review Board.

### Primary epithelial cell culture

Primary human gingival epithelial cells (PHGEC) were isolated and grown in culture as described previously [20]. Healthy gingival tissue was collected from patients undergoing oral surgery procedures, which as approved by the Institutional Review Board. Epithelial cells were cultured in flasks in keratinocyte growth medium (Keratinocytes Basal Media 2 plus growth supplements, Lonza Group Ltd., Basel, Switzerland) containing 0.1 mM calcium. Cells were cultured under three conditions, standard monolayer cultures, multilayer cultures that were undifferentiated and multilayer cultures that were differentiated by exposure to an air-liquid interface. For multilayer cultures cells (2×10^5^) were transferred to collagen-coated 1.1 cm^2^ transwell inserts and maintained with media in both upper and lower chambers until cells were confluent after ∼4 days. In differentiated multilayer cultures media was removed from the upper chamber but maintained in the bottom chamber so that cells were cultured at an air-liquid-interface for 10 days whereas media was retained for the undifferentiated multilayer cultures. Unless specifically stated multilayer cultures were differentiated. Cells were challenged with *P. gingivalis* (Pg), *S. gordonii* (Sg), or *F. nucleatum* (Fn) at 2×10^8^/cm^2^ for duration of 24 hrs unless otherwise specified.

### Bacteria and culture conditions

*Porphyromonas gingivalis* (*P. gingivalis* ATCC 33277), *F. nucleatum* (ATCC 25586) and *S. gordonii* (ATCC DL1) were purchased from the American Type Culture Collection (ATCC). *P. gingivalis* and *F. nucleatum* were cultured in modified Gifu anaerobic medium (GAM) broth (Nissui, Seiyaku Co., Ltd., Tokyo, Japan) at 37°C in an anaerobic chamber for 48 hours. *S. gordonii* was cultured anaerobically at 37°C in TSB with yeast extract (5 mg/ml) and glucose (5 mg/ml).

### Barrier test for cell cultures on inserts

*Bacteria* were added to the top chamber of transwell inserts. In some cases the protease inhibitor *leupeptin* (100 µM) was added along with bacteria. Fluorescein isothiocyanate–dextran (FITC-Dextran) 0.5 mM (Sigma-Aldrich Co. LLC, St. Louis, MO) was added to the top compartment of each transwell insert containing differentiated primary human gingival epithelial cells. Samples were removed and measured after 5 hours for fluorescent measurement on a CytoFluor 4000 plate reader (Perceptive Biosystems/Life technologies, Carlsbad, California) with 485 nm/530 nm excitation-emission filter set.

### Apoptosis Assay

*Bacteria* were added to the top chamber of transwell inserts. In some cases the protease inhibitor leupeptin (100 µM) was added along with bacteria since it inhibits *P. gingivalis* gingivains RgpA/B [11], [21]. Apoptosis was assessed by ELISA measuring cytoplasmic histone-associated- DNA fragments kit (Roche Applied Science, Indianapolis, IN) as described [22]. All experiment described were carried out a minimum of three times with similar results.

To examine FOXO1 and FOXO3 dependent apoptosis monolayer cultures of primary gingival epithelial cells were transfected with scrambled, FOXO1, or FOXO3 siRNA (5 nM) using GenMute™ siRNA transfection reagent for 6 hours. They were then incubated in keratinocyte growth medium for an additional 43 hrs and then stimulated with *P. gingivalis* overnight. As a positive control cells were incubated with 150 µM H2O2. Apoptosis was detected using DeadEnd™ Fluorometric TUNEL System (Promega Corporation, Madison, WI) and cells were counterstained with DAPI. The percent positive cells were detected with a fluorescent microscope and NIS-Elements software (Nikon Instruments Inc., Melville, NY).

### Cytokine and Keratin mRNA Levels

RNA was extracted from cells using RNeasy MiniKit and mRNA levels examined by real time PCR. Monolayer PHGEC in 6- or 24-well plates at approximately 70% confluence were transfected with FOXO1, FOXO3 and scrambled siRNA (Dharmacon, Thermo Scienific, Waltham, MA) using Genmute transfection reagent (GenMute™ siRNA Transfection Reagent, Signagen Lab) for 5 h. Cells were then rinsed and incubated in standard cell culture medium for an additional 43 hrs. Cells were then incubated with bacteria 2×10^8^/cm^2^ for 20 hrs after which they were assayed for mRNA level. Total RNA was extracted from PHGEC and assessed for mRNA gene expression of TLR-2, TLR-4, keratin-1, -10 and -14, involucrin, FOXO1, FOXO3, BID, TRADD, integrin-β1, -β3 and –β6 by real-time PCR using Roche primers and probe sets (Roche Applied Science, USA). Results were normalized to a housekeeping gene, ribosomal protein L32.

### Statistical analysis

Statistical significance was determined by one-way analysis of variance (ANOVA) for most assays. For RNAi assays significance was determined by ANOVA with Tukey's post-hoc test. Significant differences were established at P<0.05. The experiments were performed 3 times with similar results.

## Results

**Figure 1:**
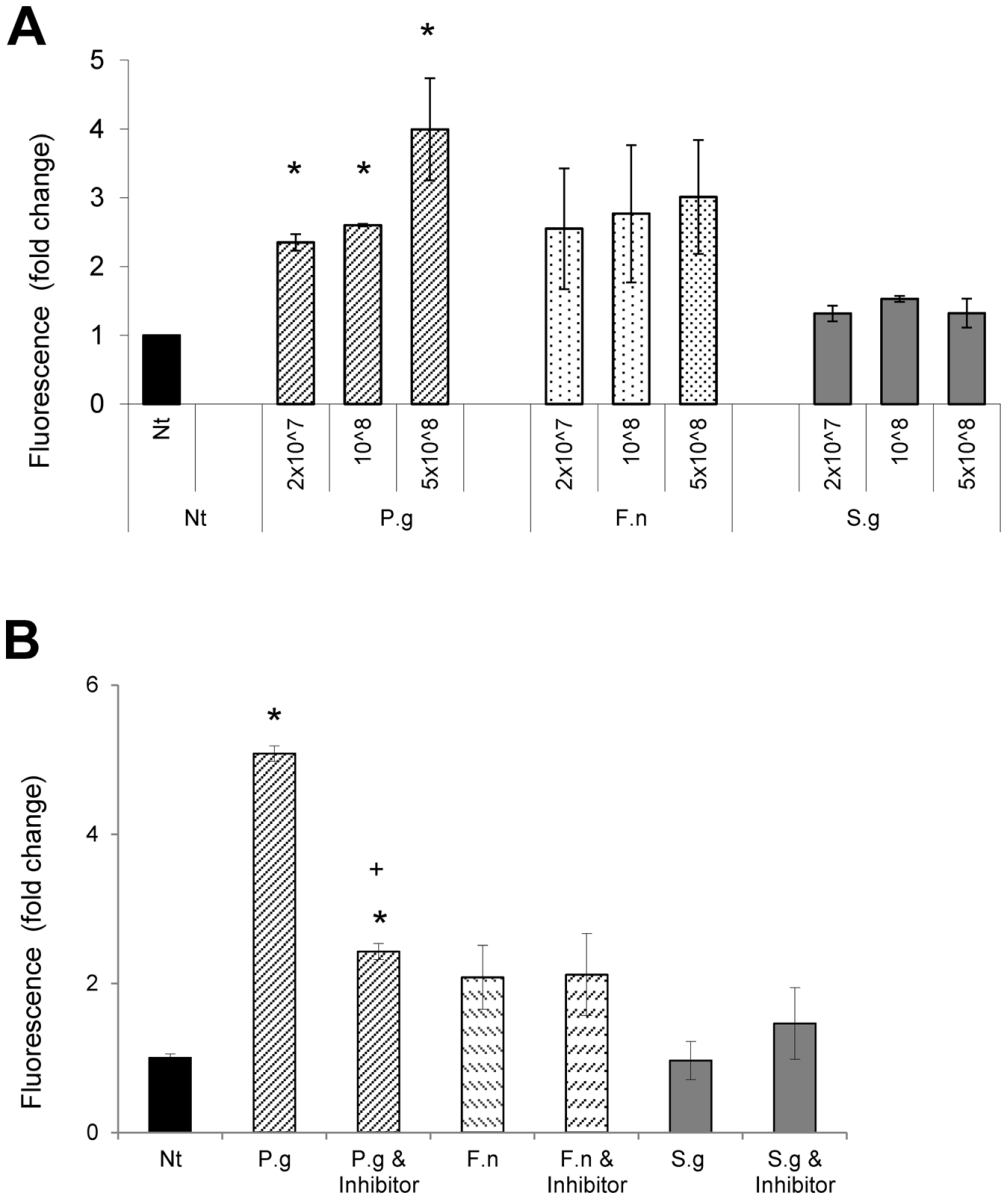
*P. gingivalis* but not *F. nucleatum* or *S. gordonii* induces loss of barrier function. *P. gingivalis*, *F. nucleatum* and *S. gordonii* were tested for loss of barrier function with fluorescein isothiocyanate–dextran (FITC-Dextran) with multi-layer, differentiated primary human gingival epithelial cells induced by air-liquid interface. A: Bacterial dose response. B: Incubation with the protease inhibitor leupeptin (100 µM). * Significantly different from matched control (P<0.05). + Significantly inhibited by leupeptin (P<0.05).

The barrier function of primary keratinocytes was measured by use of fluorescent labeled dextran (Figure 1). The amount of dextran that crossed the gingival epithelial barrier was inversely related to the concentration of *P. gingivalis* in a dose-dependent manner. *P. gingivalis* caused a 2 to 4 fold increase in barrier breakdown (P<0.05). *F. nucleatum* caused a trend toward increased breakdown but was not statistically significant (P>0.05). *S. gordonii* did not disrupt the epithelial barrier (P>0.05) (Figure 1A). To determine whether this was due to proteolytic enzymes PHGEC were incubated simultaneously with *P. gingivalis* and leupeptin (5×10^8^). *P. gingivalis* induced a 4 fold increase in loss of barrier function as measured by fluorescence intensity and half of this increase was blocked by leupeptin (P<0.05). As noted above, *F. nucleatum* and *S. gordonii* had no effect on epithelial barrier function nor was it affected by leupeptin in the absence of *P. gingivalis* (P>0.05) (Figure 1B).

**Figure 2:**
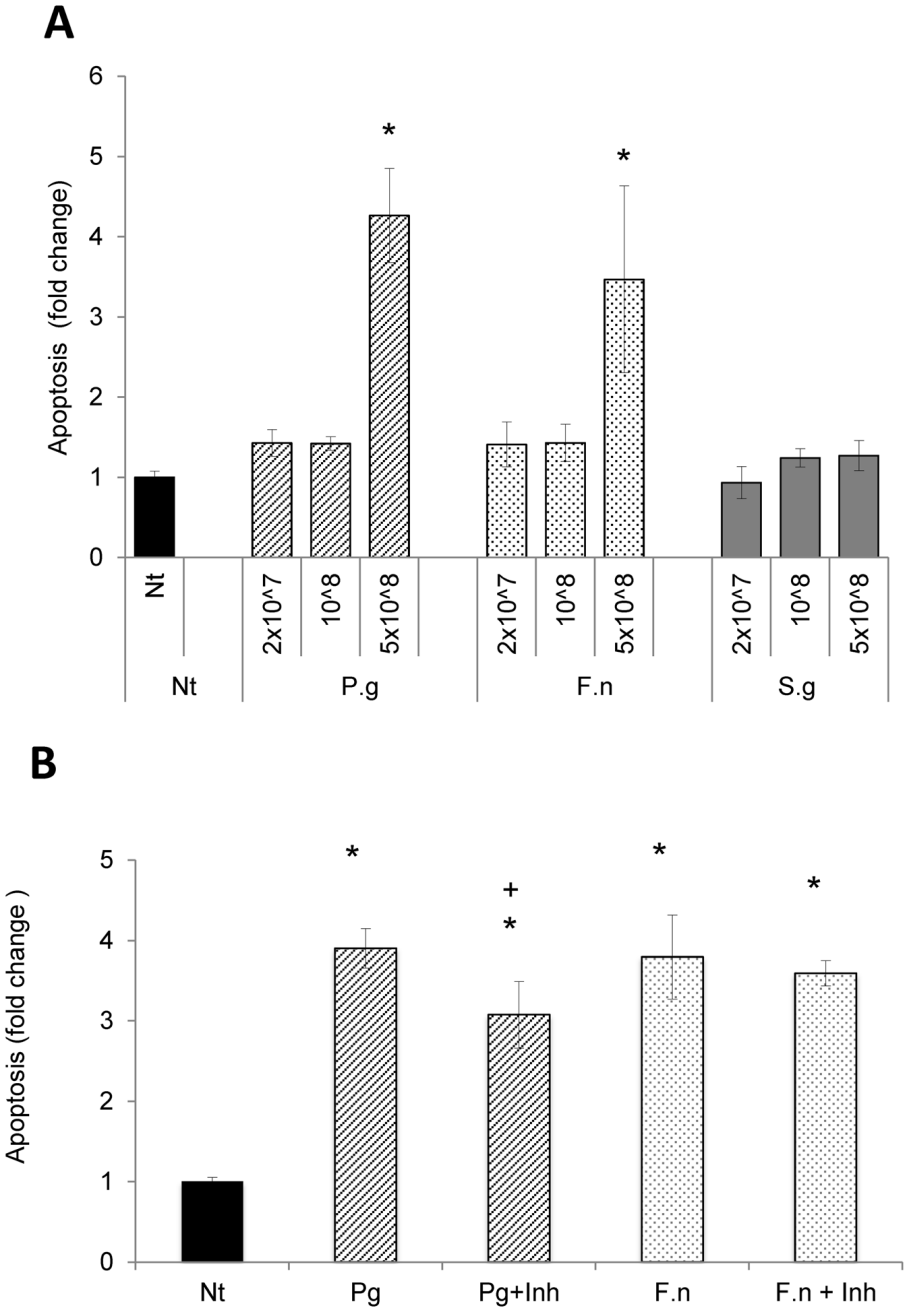
*P. gingivalis* but not *F. nucleatum* or *S. gordonii* induces apoptosis of differentiated multi-layer culture of gingival keratinocytes. *P. gingivalis*, *F. nucleatum and S. gordonii* were incubated with multi-layer, differentiated primary human gingival epithelial cells induced by air-liquid interface. Apoptosis was assessed by ELISA measuring cytoplasmic histone-associated DNA. A: Bacterial dose response. B: Incubation with the protease inhibitor leupeptin (100 µM). * Significantly different from matched control (P<0.05). + Significantly inhibited by leupeptin (P<0.05).

The effect of bacteria on three-dimensional cultures of gingival epithelial cells was examined by assessing the degree of apoptosis that each induced (Figure 2). *P. gingivalis* and *F. nucleatum* both stimulated a 3 to 4 fold increase in apoptosis which was statistically significant at 5×10^8^ bacteria. *S. gordonii* had no effect on apoptosis (P>0.05). To determine whether gingipains played a prominent role in apoptosis, cells were co-incubated with bacteria and leupeptin. Leupeptin reduced *P. gingivalis* stimulated apoptosis by 20%, which was significant (P<0.05) but had no effect on *F. nucleatum* stimulated apoptosis. Thus gingipains contributed but did not play a predominant role in *P. gingivalis* induced apoptosis of multi-layer cultures and had no effect on *F. nucleatum* induced apoptosis.

**Figure 3:**
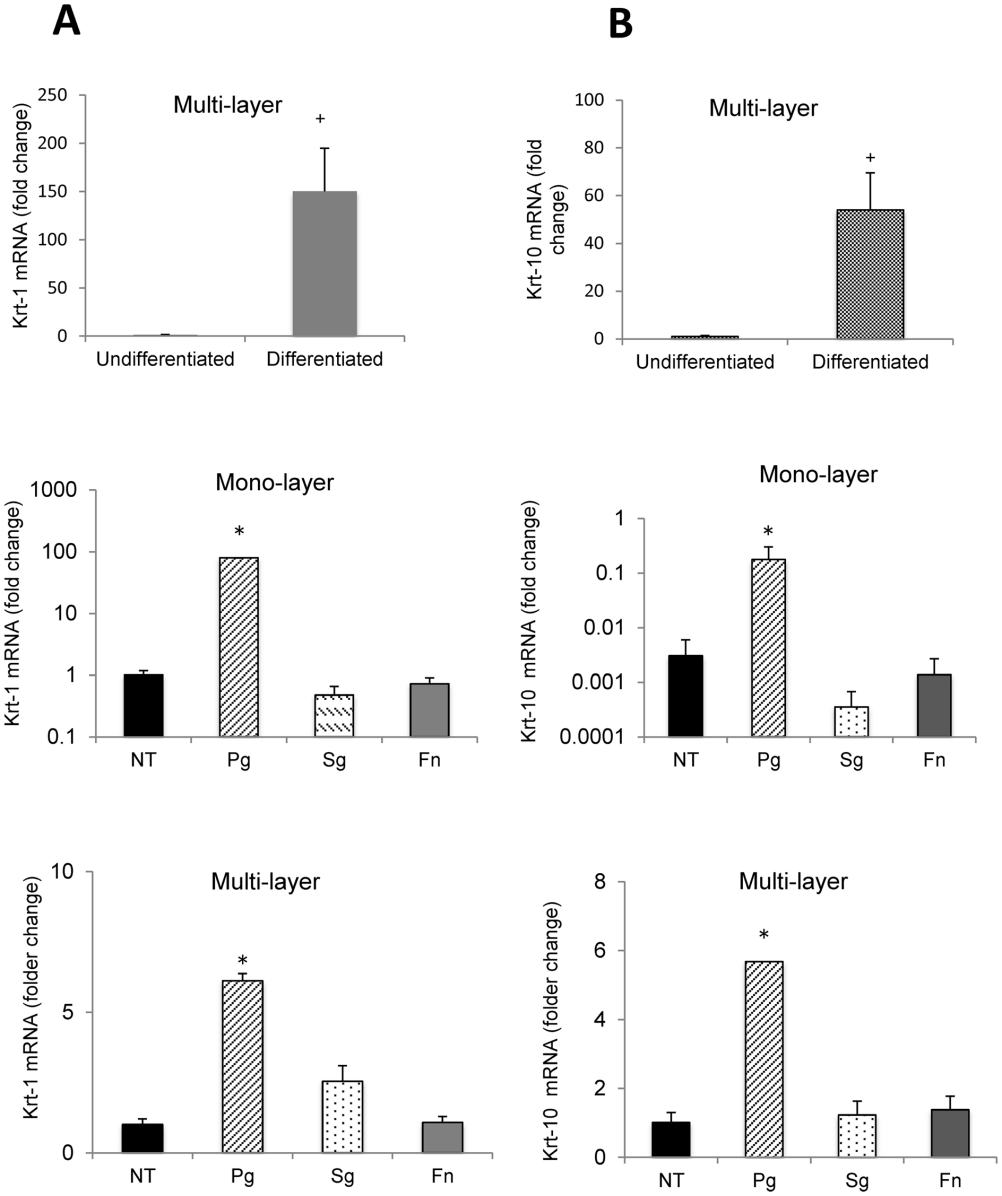
*P. gingivalis* but not *F. nucleatum* or *S. gordonii* induces expression of differentiation markers. Primary human gingival epithelial cell cultures were incubated with or without exposure to an air-liquid-interface to induce differentiation and then challenged with *P. gingivalis* (Pg), S. *gordonii* (Sg), or *F. nucleatum* (Fn) at 2×10^8^/cm^2^ for 24 hrs. mRNA levels of keratin-1 or keratin-10 were measured by real-time PCR. +Significant difference between undifferentiated and differentiated cells (P<0.05). * Significantly different from matched control (P<0.05).

The expression of keratin-1 and -10 reflects the level of differentiation with both increasing as differentiation occurs. mRNA levels of keratin-1 and -10 increased 50 to 100 fold when differentiation was stimulated in PHGEC when exposed to an air-liquid interface (Figure 3A). This increase occurred within one week of culture. When undifferentiated PHGEC were challenged with *P. gingivalis* in the absence of an air-liquid interface a ∼100 fold increase in keratin-1 was stimulated. *P gingivalis* induced a 6 fold increase in keratin-1 and -10 mRNA levels in multi-layered, well-differentiated PGHEC (P<0.05). Similar results were obtained with keratin-10 (Figure 3B). In contrast, *F. nucleatum* and *S. gordonii* had little effect on expression of these keratinocyte differentiation markers (P>0.05) (Figure 3A&B).

**Figure 4:**
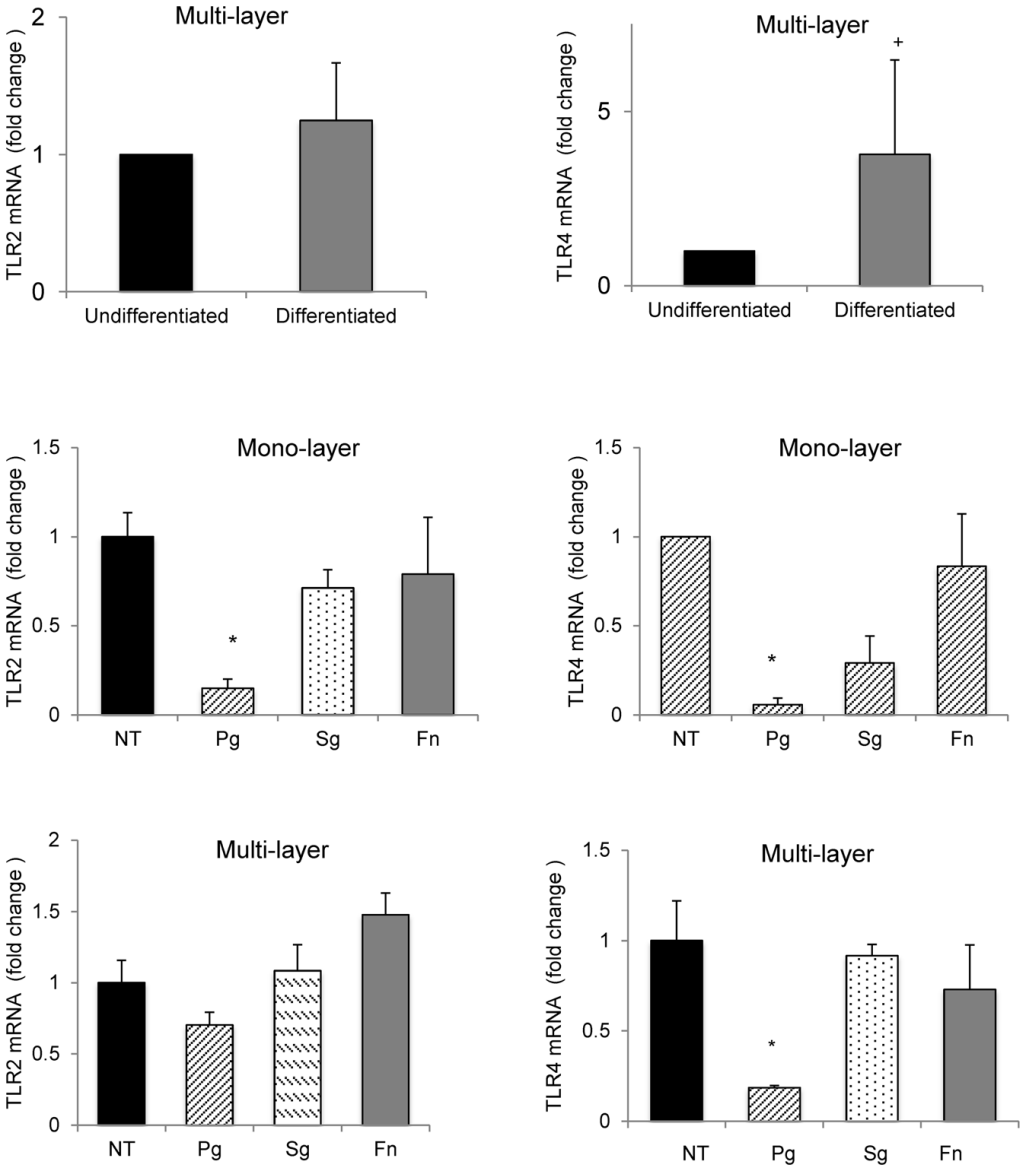
*P. gingivalis* but not *F. nucleatum* or *S. gordonii* down-regulates TLR2 and TLR4 mRNA levels. Primary human gingival epithelial cell cultures were incubated with or without exposure to an air-liquid-interface to induce differentiation and then challenged with *P. gingivalis* (Pg), S. *gordonii* (Sg), or *F. nucleatum* (Fn) at 2×10^8^/cm^2^ for 20 hrs. mRNA levels of TLR-2 or TLR-4 were measured by real-time PCR. +Significant difference between undifferentiated and differentiated cells (P<0.05). * Significantly different from matched control (P<0.05).

Since TLR-4 and TLR-2 are important host response genes we examined their mRNA levels as a function of differentiation status (Figure 4). The formation of a multi-layer culture with induced keratinocyte differentiation stimulated a significant increase in TLR-4 (P<0.05) but not TLR-2 (P>0.05) mRNA levels. *P. gingivalis* caused a 80–95% decrease in TLR-4 mRNA levels in both mono- and multi-layer cultures (P<0.05). In monolayer cultures *P. gingivalis* induced an 85% decrease in TLR-2 mRNA levels (P<0.05) while in multi-layer differentiated cells it did not have a significant effect on TLR-2 mRNA levels (P>0.05). Thus, the differentiation status of the cells affected TLR-4 but not TLR-2 mRNA levels and their induction by *P. gingivalis*. In contrast to *P. gingivalis*, *F. nucleatum* and *S. gordonii* had no significant effect on TLR-2 or TLR-4 mRNA levels (P>0.05) (Figure 4).

**Figure 5:**
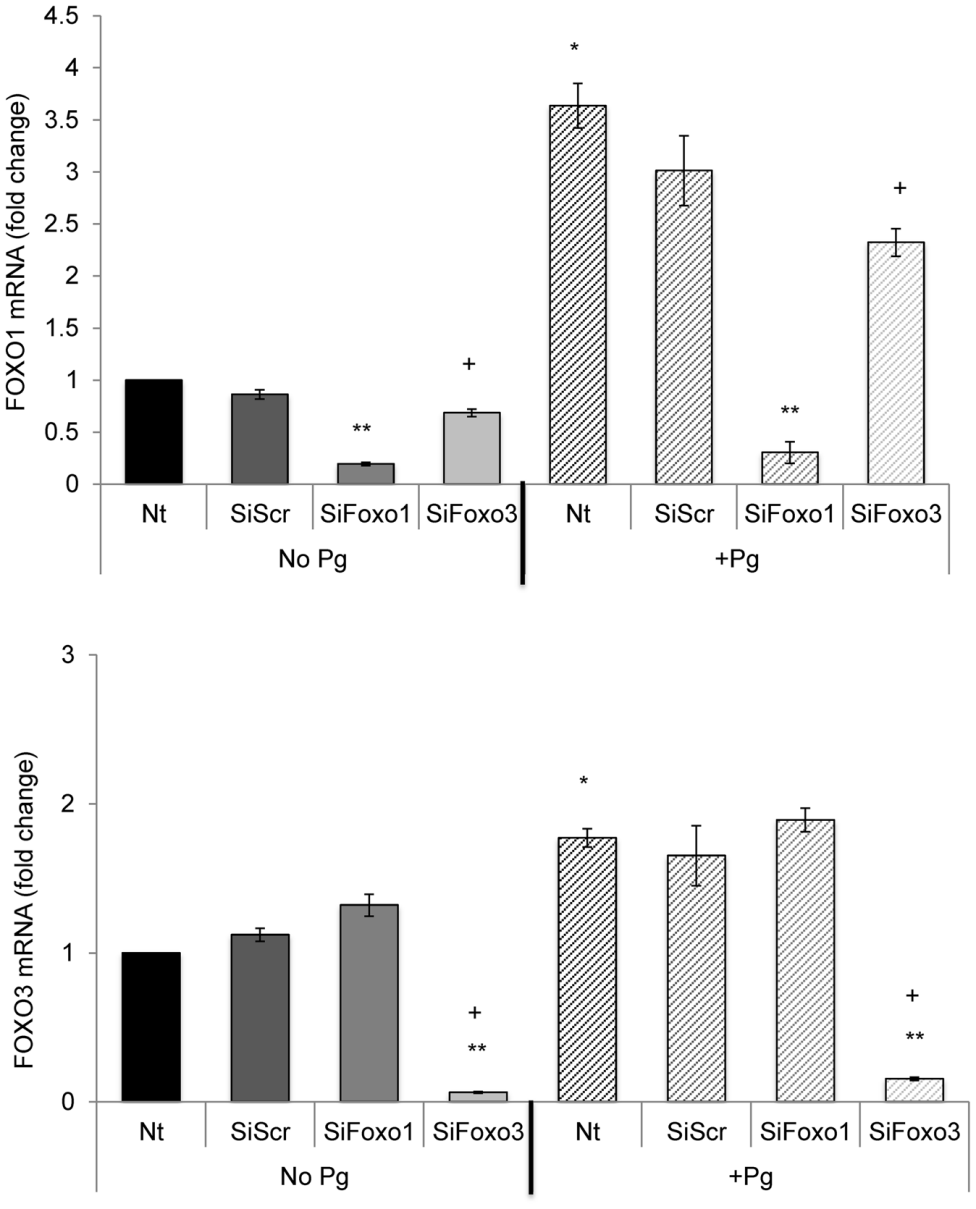
*P. gingivalis* up-regulates FOXO1 and FOXO3 mRNA levels. Primary human gingival epithelial cell cultures were incubated with or without *P. gingivalis* at MOI = 1∶50 for 20 hrs. In some cases cells were pre-incubated with FOXO1 siRNA (SiFOXO1), FOXO3 siRNA (SiFOXO3) or scrambled siRNA (SiScr) FOXO1 prior to stimulation with bacteria. FOXO1 and FOXO3 mRNA levels were measured by real-time PCR. * Significantly different from control cells without bacterial stimulation (P<0.05). ** Significantly different between scrambled siRNA and FOXO1 or FOXO3 siRNA (P<0.05). + Significantly different between FOXO1 siRNA and FOXO3 siRNA (P<0.05).

Based on the above results we examined monolayer cultures to better understand how *P. gingivalis* affects keratin and TLR mRNA levels and key genes involved in apoptosis and barrier function. This was accomplished by investigating the capacity of *P. gingivalis* to stimulate FOXO1 and FOXO3 mRNA levels and to examine the functional role of these two key transcription factors by RNAi (Figure 5). *P. gingivalis* stimulated a 3.5 fold increase in FOXO1 mRNA and a 2-fold increase in FOXO3 mRNA levels, which were significant (P<0.05). Moreover, siRNA to FOXO1 reduced FOXO1 mRNA levels by approximately 80% compared to scrambled siRNA (P<0.05) without affecting FOXO3 mRNA levels (P>0.05) and FOXO3 siRNA reduced FOXO3 mRNA levels by 90% compared to scrambled siRNA (P<0.05) without down-regulating FOXO1 mRNA (Figure 5). siRNA was also shown to effectively knockdown FOX01 at the protein level as determined by immunofluorescence (data not shown). FOXO1 siRNA and FOXO3 siRNA both blocked *P. gingivalis* enhanced FOXO1 and FOXO3 mRNA levels, respectively, while scrambled siRNA had no effect.

**Figure 6:**
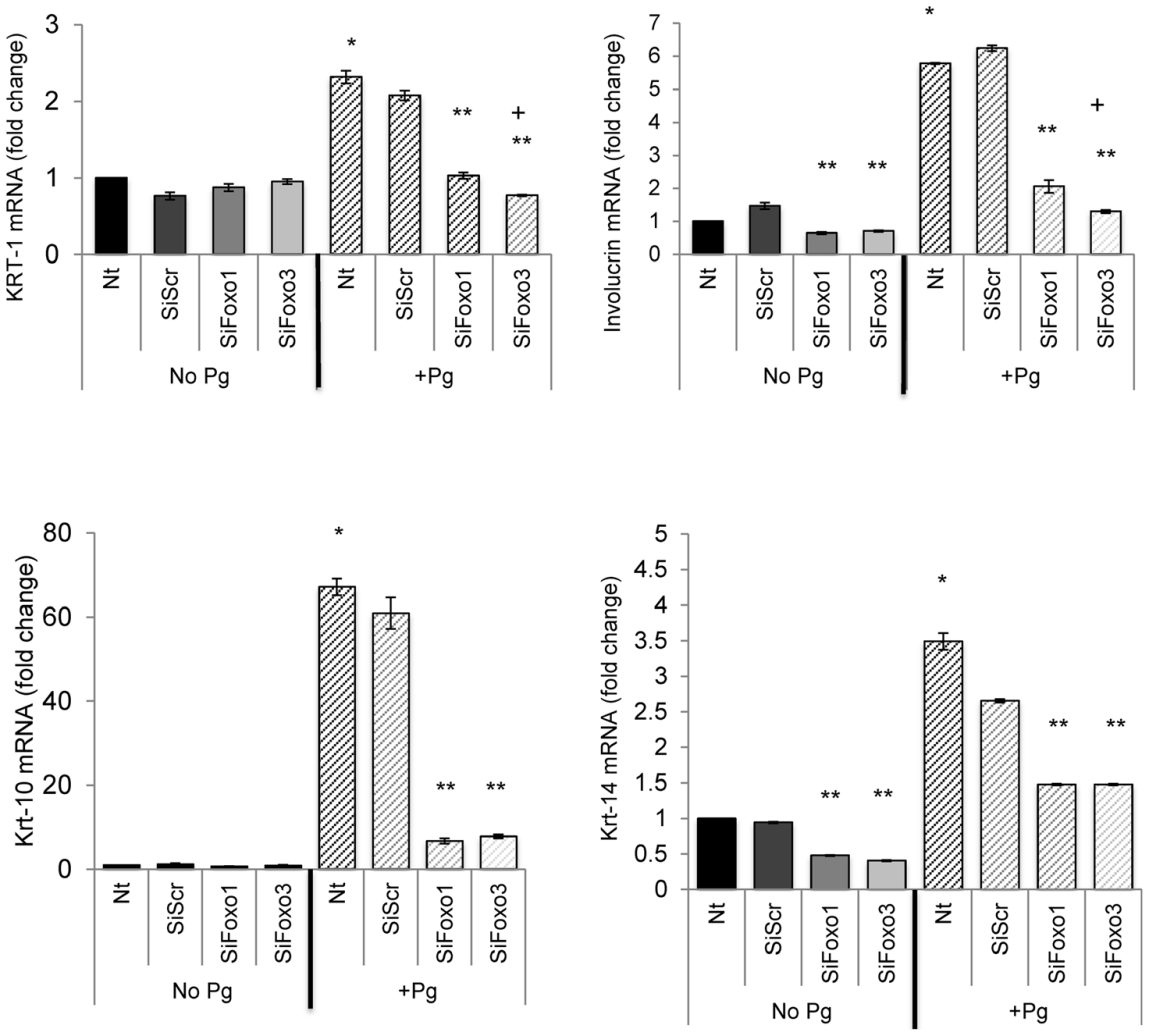
*P. gingivalis* up-regulation of keratinocyte differentiation markers is FOXO1 or FOXO3 dependent. Primary human gingival epithelial cell cultures were incubated with or without *P. gingivalis* at MOI = 1∶50 for 20 hrs. In some cases cells were pre-incubated with FOXO1 siRNA (SiFOXO1), FOXO3 siRNA (SiFOXO3) or scrambled siRNA (SiScr) FOXO1 prior to stimulation with bacteria. Real-time PCR was used to measure mRNA levels of keratin-1, keratin-10, involucrin and keratin-14. * Significantly different from control cells without bacterial stimulation (P<0.05). ** Significantly different between scrambled siRNA and FOXO1 or FOXO3 siRNA (P<0.05). + Significantly different between FOXO1 siRNA and FOXO3 siRNA (P<0.05).

The effect of FOXO1 knockdown on mRNA levels of differentiation markers, keratin-1, -10, -14 and involucrin was measured (Figure 6). The basal levels of keratin-1 and -10 were not affected by FOXO1 siRNA and FOXO3 siRNA (P>0.05). The basal levels of involucrin and keratin-14 were reduced 34% to 52% by FOXO1 siRNA (P<0.05) and 30% to 60% by FOXO3 siRNA (P<0.05), respectively. The capacity of *P. gingivalis* to increase mRNA levels of keratin-1 and -10 was reduced by 50% to 89% by FOXO1 siRNA and by 63% to 87% by FOXO3 siRNA compared to control siRNA (P<0.05). The capacity of *P. gingivalis* to stimulate mRNA levels of keratin-14 and involucrin was similarly reduced by knockdown of FOXO1 and FOXO3 by between 20% and 45% (Figure 6).

**Figure 7:**
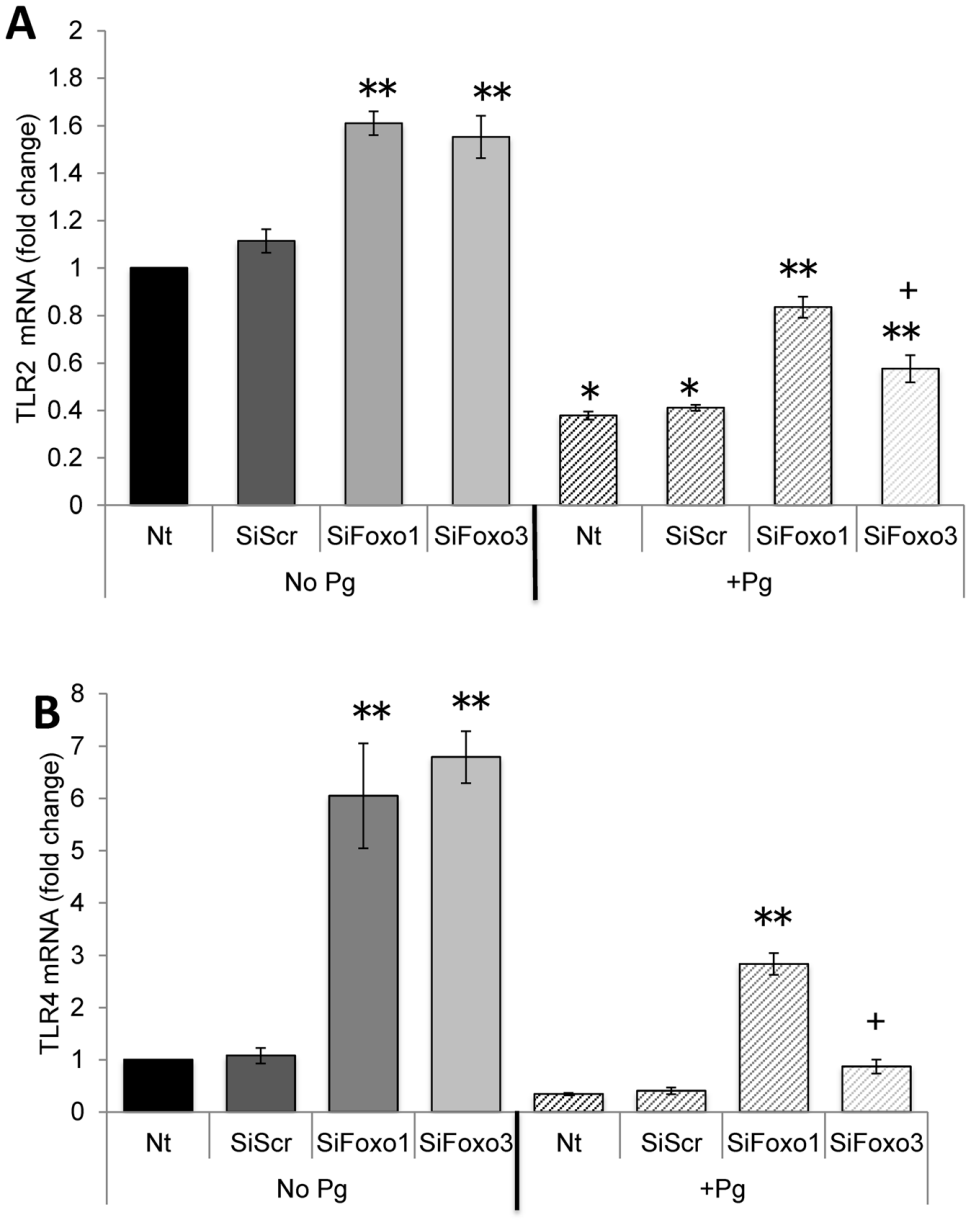
FOXO1 and FOXO3 regulate basal and *P. gingivalis* up-regulated mRNA levels of TLR2 and TLR4. Primary human gingival epithelial cell cultures were incubated with or without *P. gingivalis* at MOI = 1∶50 for 20 hrs. In some cases cells were pre-incubated with FOXO1 siRNA (SiFOXO1), FOXO3 siRNA (SiFOXO3) or scrambled siRNA (SiScr) FOXO1 prior to stimulation with bacteria. Real-time PCR was used to measure mRNA levels of TLR-2 and TLR-4. * Significantly different from control cells without bacterial stimulation (P<0.05). ** Significantly different between scrambled siRNA and FOXO1 or FOXO3 siRNA (P<0.05). + Significantly different between FOXO1 siRNA and FOXO3 siRNA (P<0.05).

Whether mRNA levels of TLR-2 or TLR-4 are FOXO dependent was also examined (Figure 7). The basal expression of both TLR-2 and TLR-4 was enhanced 1.5 times to 5.6 times by FOXO1 (P<0.05) and 1.4 times to 6.3 times by FOXO3 knockdown (P<0.05) compared to scrambled siRNA, suggesting that FOXO1 and FOXO3 both act to suppress TLR-2 and TLR-4 mRNA levels. The capacity of *P. gingivalis* to down-regulate TLR-2 was reduced by 50% with FOXO1 knockdown and by 30% with FOXO3 knockdown compared to control siRNA (Figure 7A). Similarly, the capacity of *P. gingivalis* to down-regulate TLR-4 mRNA levels was reduced 85% with FOXO1 siRNA and 55% with FOXO3 siRNA compared to control siRNA (P<0.05) (Figure 7B). Thus FOXO1 and FOXO3 suppressed basal mRNA levels of TLR2 and TLR4 and the impact of *P. gingivalis* on TLR-2 and TLR-4 levels was mediated in part by FOXO1 and FOXO3.

**Figure 8:**
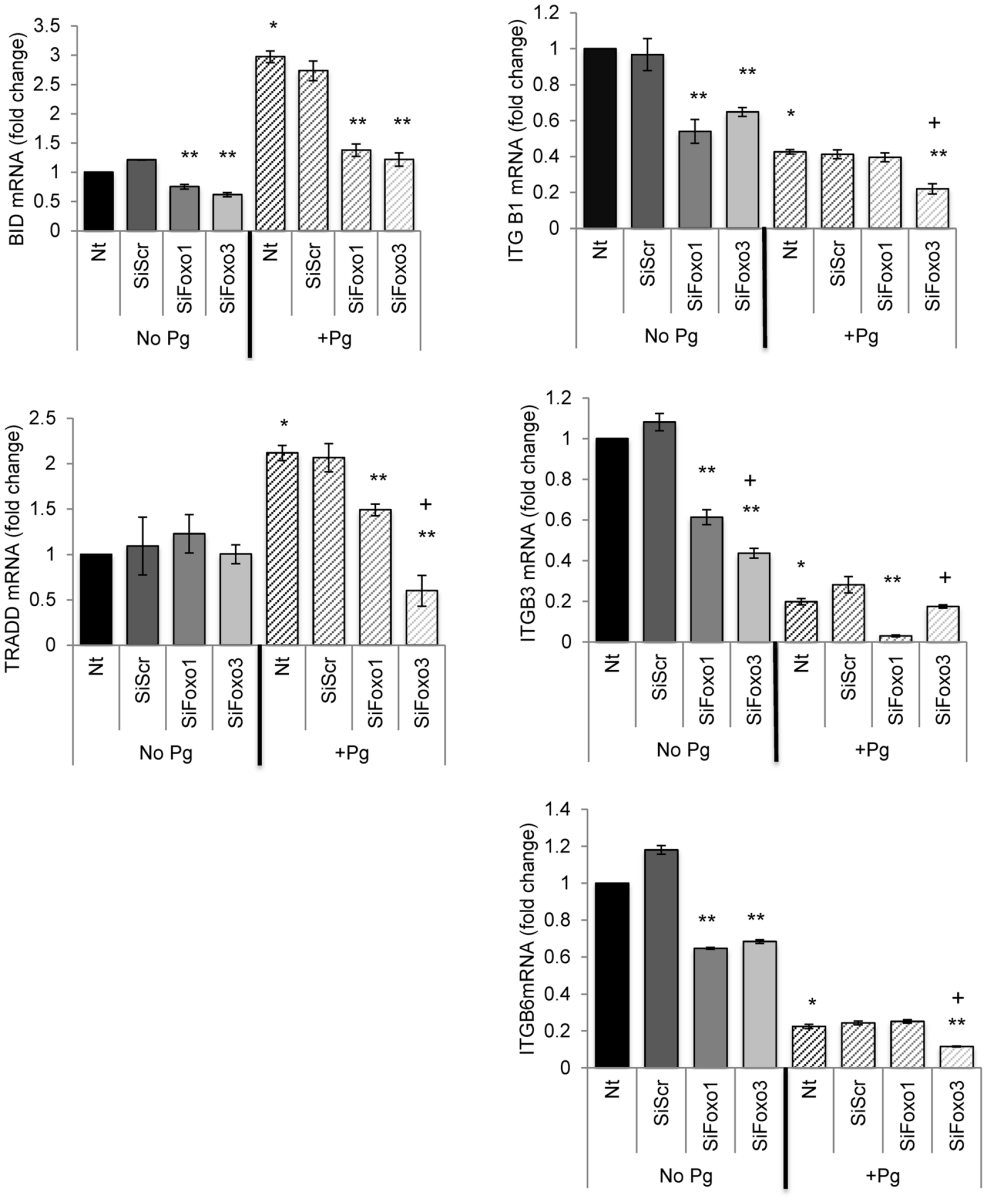
*P. gingivalis* modulates mRNA levels of apoptotic and barrier function genes through FOXO1 or FOXO3. Primary human gingival epithelial cell cultures were incubated with or without *P. gingivalis* at MOI = 1∶50 for 20 hrs. In some cases cells were pre-incubated with FOXO1 siRNA (SiFOXO1), FOXO3 siRNA (SiFOXO3) or scrambled siRNA (SiScr) FOXO1 prior to stimulation with bacteria. mRNA levels of apoptotic genes (BIDD and TRAD) and integrins that affect barrier function (beta-1, beta-3 and beta-6) were measured by real-time PCR. * Significantly different from control cells without bacterial stimulation (P<0.05). ** Significantly different between scrambled siRNA and FOXO1 or FOXO3 siRNA (P<0.05). + Significantly different between FOXO1 siRNA and FOXO3 siRNA (P<0.05).

Several integrins participate in epithelial barrier function [23]. Integrin beta-1, -3 and -6 basal mRNA levels were dependent upon FOXO1 and FOXO3 since all three were reduced approximately 50% with FOXO1 or FOXO3 knockdown (P<0.05) (Figure 8). *P. gingivalis* decreased beta-1, -3 and -6 mRNA levels by approximately 57% to 80% compared to untreated group (P<0.05). The capacity of *P. gingivalis* to downregulate mRNA levels of integrin beta-3 was reduced 89% by FOXO1 siRNA (P<0.05) but not by knockdown of FOXO3 (P>0.05). Moreover, the ability of *P. gingivalis* to reduce integrin beta-1 and beta-6 mRNA levels was reduced ∼50% by FOXO3 siRNA (P<0.05) but not FOXO1 siRNA (P>0.05) (Figure 8).

We also examined mRNA levels of BID and TRADD (Figure 8), which are pro-apoptotic genes that have previously been shown to be induced by *P. gingivalis* [24]. Basal levels of BID mRNA were reduced approximately 50% by FOXO1/FOXO3 knockdown (P<0.05). The capacity *P. gingivalis* to increase BID mRNA levels was largely blocked by FOXO1 and FOXO3 knockdown (P<0.05).

**Figure 9:**
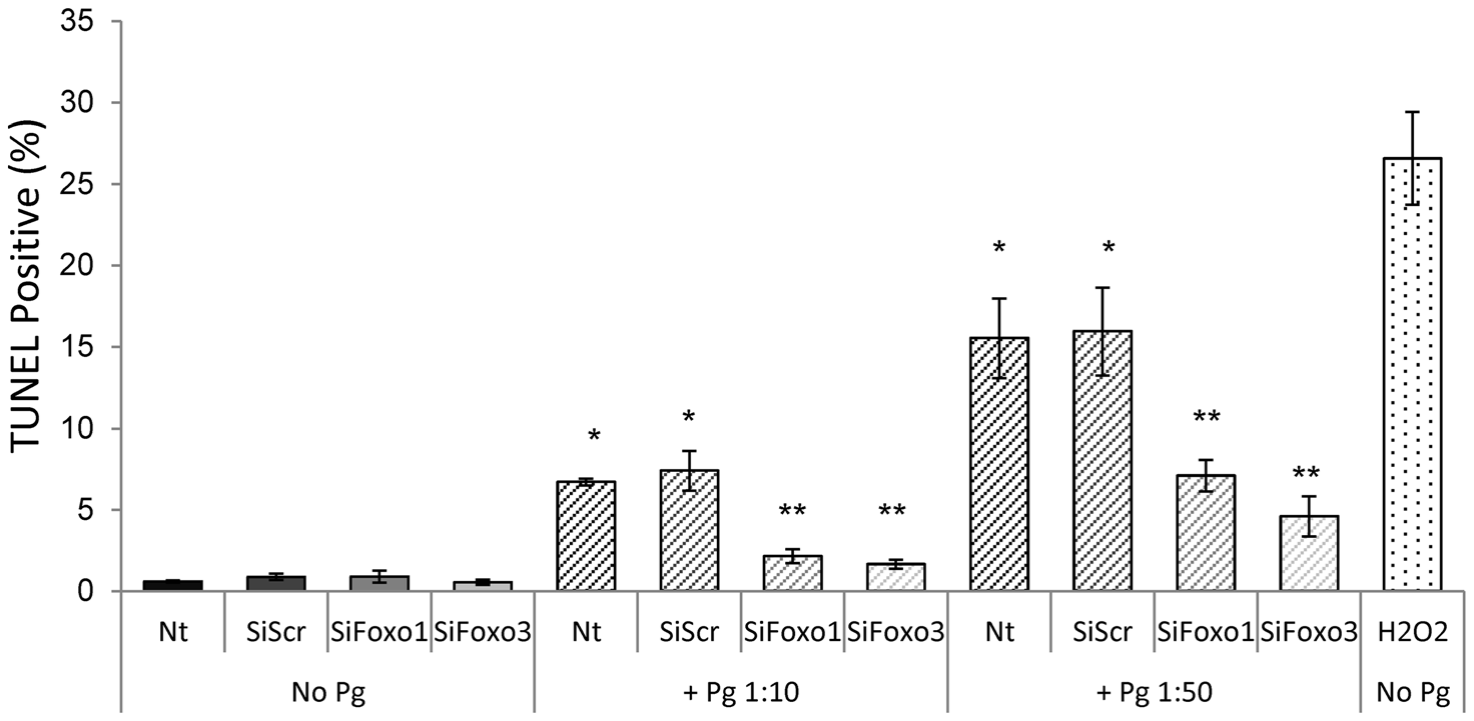
*P. gingivalis* induces gingival epithelial cell apoptosis through FOXO1 or FOXO3. Primary human gingival epithelial cells were incubated with or without P. gingivalis at MOI = 1∶10 or 1∶50 for overnight. In some cases cells were pre-incubated with FOXO1 siRNA (SiFOXO1), FOXO3 siRNA (SiFOXO3) or scrambled siRNA (SiScr) prior to incubation with bacteria. Apoptotic cells were assessed by the TUNEL assay. * Significantly different from control cells with bacterial stimulation (P<0.05). ** Significantly different between scrambled siRNA and FOXO1 or FOXO3 siRNA (P<0.05).

To link FOXO1 and FOX3 dependent changes in apoptotic gene expression with a functional assay, the effect of *P. gingivalis* on keratinocyte apoptosis was measured by the TUNEL assay. The results indicate the P. gingivalis stimulated apoptosis of primary gingival epithelial cells. Knockdown of FOXO1 or FOXO3 significantly reduced the percent apoptotic gingival epithelial cells induced by *P. gingivalis* by 55–70% (P<0.05) with no difference between FOXO1 and FOXO3 siRNA (Fig. 9).

## Discussion

We examined the impact of *P. gingivalis* on differentiated gingival epithelial cells and on key genes that regulate key aspects of keratinocyte function. *P. gingivalis*, but not *F. nucleatum* and *S. gordonii* disrupted the capacity of these cells to form a diffusion barrier that was partially gingipain dependent. Both *P. gingivalis* and *F. nucleatum* induced apoptosis of three dimensional gingival epithelial cultures with gingipains playing a minor role in *P. gingivalis* induced apoptosis. *P. gingivalis* generally reduced mRNA levels of important host defense components TLR-2, TLR-4 in both differentiated and undifferentiated cultures of primary human gingival epithelial cells while *F. nucleatum* and *S. gordonii* generally did not. Interestingly, *P. gingivalis* stimulated keratin 1 and keratin 10 mRNA levels in undifferentiated gingival keratinocytes suggesting that it promotes differentiation. To investigate mechanistically the effect of *P. gingivalis* we demonstrated that *P. gingivalis* stimulated FOXO1 and FOXO3 mRNA levels, keratin-1, keratin-10, keratin-14, involucrin mRNA that is associated with differentiation of keratinocytes and BID and TRADD mRNA that modulates apoptosis. In contrast, *P. gingivalis* down-regulated mRNA levels of the host-response genes TLR-2 and -4 and integrins involved in barrier function, beta-1, -3 and -6. The role of FOXO1 or FOXO3 in modulating the mRNA levels of host target genes affected by bacteria has received relatively little attention and may be clinically important. Interestingly basal mRNA levels of several key target genes were dependent upon FOXO1 as evidence by decreased basal levels of involucrin, keratin-14, BID, TRADD, integrins beta-1, -3, and -6 when FOXO1 or FOXO3 were knocked down and increased mRNA levels of TLR-2 and TLR-4 when FOXO1 or FOX3 was silenced. Thus, FOXO1 or FOXO3 may play an important role in mucosal epithelium by regulating the mRNA levels of key target genes. This is supported by findings that the capacity of P. gingivalis to stimulate an increase in mRNA levels of pro-apoptotic genes and for P. gingivalis to stimulate apoptosis in gingival epithelial cells are both FOXO1 and FOXO3 dependent.

Oral keratinocytes form various cellular contacts, including tight junctions, and thus are able to create an epithelial barrier. It has previously been reported that *P. gingivalis* disrupts barrier function of Madin-Darby canine kidney (MDCK) cells in a process that was thought to be dependent upon protease activity but was not proven [25]. Our findings suggest that some but not all of *P. gingivalis* disruption of epithelial barrier in multilayers is inhibited by leupeptin which blocks cysteine, serine and threonine peptidases. The partial effect may be due to not blocking lysine-specific (Lys-X) gingipains produced by *P. gingivalis* [26]. Moreover the partial effect of blocking *P. gingivalis* gingipains is consistent with a previous report that protease inhibitors reduce *P. gingivalis* disruption of epithelial barrier function but do not block it [27]. In addition, we found that *P. gingivalis* down-regulated mRNA levels of beta integrins that play a role in barrier function. These results contrast with a recent report that *P. gingivalis* does not inhibit barrier function [12]. The explanation for the differences in results is not readily apparent.

Although viruses are well known to degrade cellular mRNA message [28] down-regulation of host mRNA by bacteria is less well known. Host mRNA degradation may happen directly by several mechanisms including up-regulation of miRNA in host cells that silence target mRNA degradation [29]. Bacteria may down regulate host genes as a survival strategy. We identify here that *P. gingivalis* can activate FOXO transcription factors that negatively regulate TLR-2 and TLR-4 transcript levels, both of which have been shown to mediate the response to *P.* gingivalis [30]. The down-regulation of TLR-2 and -4 may reflect a mechanism through which the inflammatory response to bacteria is attenuated in oral epithelial cells. In addition we found that knockdown of FOXO1 and FOXO3 enhanced endogenous TLR-2 and TLR-4 mRNA levels suggesting that FOXO1/3 suppress TLR-2 and TLR-4. Interestingly, P. gingivalis increased FOXO1and the capacity P. gingivalis to suppress TLR-2/4 was partially reduced when FOXO1 in particular was silenced. This suppression of TLR-2 and 4 by FOXO1/3 represents a previously unrecognized function of these FOXO transcription factors.

*P. gingivalis* modulates several aspects of epithelial cell biology including progression through the cell cycle and apoptosis [13], [31], [32]. A recent report suggests that *P. gingivalis* has no effect on apoptosis of multilayer keratinocytes after 24 hours [12]. Our results suggest that *P. gingivalis* does induce apoptosis in this period of time in well differentiated multilayers cultures of oral epithelial cells and that gingipains play a minor although statistically significant role in this process. *P. gingivalis* has been shown to have various apoptotic or anti-apoptotic effects depending upon the cell type, the time frame examined, the strain of P. gingivalis used and may also influence the effect of other pro-apoptotic factors [33]. In addition we found that *P. gingivalis* enhanced differentiation of these cells as shown by enhanced mRNA levels of involucrin and keratin-1, -10 and 14, which are markers of differentiated epithelial cells [2], [34], [35]. The fact that bacteria may modulate the differentiation status could have ramifications in a number of different situations. One is that the expression of anti-bacterial factors in keratinocytes depends upon a differentiated phenotype so that the presence of bacteria such as *P. gingivalis* may enhance the ability to respond by production of defensins [36].

To investigate whether FOXO1 or FOXO3 regulate key markers of keratinocyte differentiation we examined their impact on basal as well as *P. gingivalis* mediated keratin-1, keratin-10 and involucrin This is based on findings in other cell types that the FOXO1 transcription factors affect proliferation and differentiation [16]. We found that the basal levels of involucrin and keratin-14 were reduced when FOXO1 or FOX03 were silenced by siRNA and that the capacity of *P. gingivalis* to enhance mRNA levels of keratin-1, -10 and involucrin was FOXO1 and FOXO3 dependent. This identifies the FOXO1/FOXO3 transcription factors as regulators of keratinocyte behavior and as mediators in the response to bacteria such as *P. gingivalis*.
